# Laparoscopic transposition for crossing vessels (vascular hitch) in pure extrinsic pelvic-ureteric junction obstruction: a successful case report of a 2-year-old infant with horseshoe kidney

**DOI:** 10.1186/s40792-021-01190-y

**Published:** 2021-04-23

**Authors:** Satoshi Ieiri, Kouji Nagata

**Affiliations:** 1 Department of Pediatric Surgery, Research Field in Medical and Health Sciences, Medical and Dental Area, Research and Education Assembly, Kagoshima University, 8-35-1, Sakuragaoka, Kagoshima, 890-8520 Japan; 2 Department of Pediatric Surgery, Faculty of Medical Sciences, Kyushu University, Fukuoka, Japan

**Keywords:** Hydronephrosis, Pelvi-ureteric junction obstruction, Crossing vessels, Extrinsic obstruction, Laparoscopic surgery, Vascular hitch, Horseshoe kidney

## Abstract

**Background:**

Pediatric hydronephrosis induced by pelvic-ureteric junction obstruction (PUJO) is treated by dismembered pyeloplasty (DP) via open and laparoscopic surgery. The etiology of PUJO involves both intrinsic stenosis and extrinsic compression of crossing vessels (CVs). PUJO owing to CVs is also treated by DP, as there is no consensus concerning this vascular condition. We encountered a 2-year-old infant with pure extrinsic PUJO combined with horseshoe kidney who successfully underwent laparoscopic transposition for CVs (vascular hitch).

**Case presentation:**

A 2-year-old boy was prenatally diagnosed with left multicystic dysplastic kidney (MDCK) and right hydronephrosis and received a definitive diagnosis after birth. At 6 months old, renal scintigraphy revealed a non-functioning pattern in the left kidney and an obstructive pattern in the right, showing no response to furosemide loading. The patient also had recurrent urinary tract infection, and his right hydronephrosis gradually worsened. We decided to perform surgery for the right PUJO. Preoperative enhanced computed tomography detected three right renal vessels independently branching from the abdominal aorta. The middle renal vessels were located at the ventral side of the pelvis and coincident with the site of PUJO. These vessels were suspected of being CVs. The patient underwent laparoscopic surgery electively. A 5-mm trocar was inserted at the umbilicus for a 5-mm, 30° rigid scope. Two additional ports were then inserted under laparoscope inspection. The dilated right pelvis and CVs were detected after ascending colon mobilization. To confirm the pathogenesis of PUJO, the CVs were dissected and taped. After taping the CVs, an intraoperative diuretic test was performed using furosemide loading. Peristalsis of the right ureter was recognized, and the extrinsic PUJO owing to the CVs was definitively confirmed. We therefore performed transposition for the CVs (vascular hitch procedure). The CVs were mobilized in the cranial direction and those were wrapped by dilated pelvis. The post-operative course was uneventful. The renal scintigraphy findings improved and showed a favorable response of furosemide loading.

**Conclusions:**

The laparoscopic vascular hitch procedure is minimally invasive and effective for extrinsic PUJO due to CVs. Anastomotic stricture after Anderson and Hynes DP can be prevented by appropriate patient selection.

**Supplementary Information:**

The online version contains supplementary material available at 10.1186/s40792-021-01190-y.

## Background

Pediatric hydronephrosis induced by pelvic-ureteric junction obstruction (PUJO) has been treated by dismembered pyeloplasty (DP) via open and laparoscopic surgery since the procedure was first described by Anderson and Hynes [1, 2]. Anderson and Hynes DP (AHDP) is thus an established procedure and frequently performed for PUJO in children and adult patients [3]. Recently, robotic surgery was also introduced for this procedure [4]. The etiology of PUJO involves both intrinsic stenosis and extrinsic compression of crossing vessels (CVs) [5]. However, while PUJO due to CVs is frequently recognized in adult patients, it is rarely found in neonates and infants [6]. PUJO owing to CVs is also usually treated by AHDP because of a lack of a consensus concerning this vascular condition [7].

Displacing lower-pole CVs for extrinsic PUJO (vascular hitch [VH]) was first described by Hellstrom and modified by Chapman [8, 9]. VH is highly effective and a relatively low-invasive procedure, depending on the case, but the indications and outcomes in children are unclear and controversial [10].

We herein report a 2-year-old infant who successfully underwent laparoscopic transposition for CVs in pure extrinsic PUJO combined with horseshoe kidney (HSK).

## Case presentation

A 2-year-old boy had received a prenatal diagnosis of left MDCK and right hydronephrosis. The patient was delivered at 39 weeks, with a body weight of 2910 g, and then received a definitive diagnosis of left MCDK and right hydronephrosis (grade I) after birth. The renal function of the left MCDK with a thin cortex had been almost entirely ablated, but the right kidney was suspected to have adequately compensated for the left MCDK. The patient also had a chromosomal abnormality (46, XY, der(5)t(5;13)(p15.3;q22)), atrial septal defect and mental retardation.

**Fig. 1:**
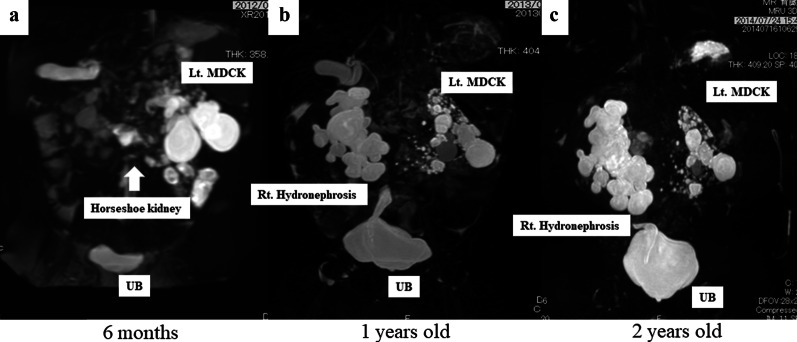
Preoperative MR urography findings. **a** 3 months old, **b** 1 years old, **c** 2 years old

**Fig. 2:**
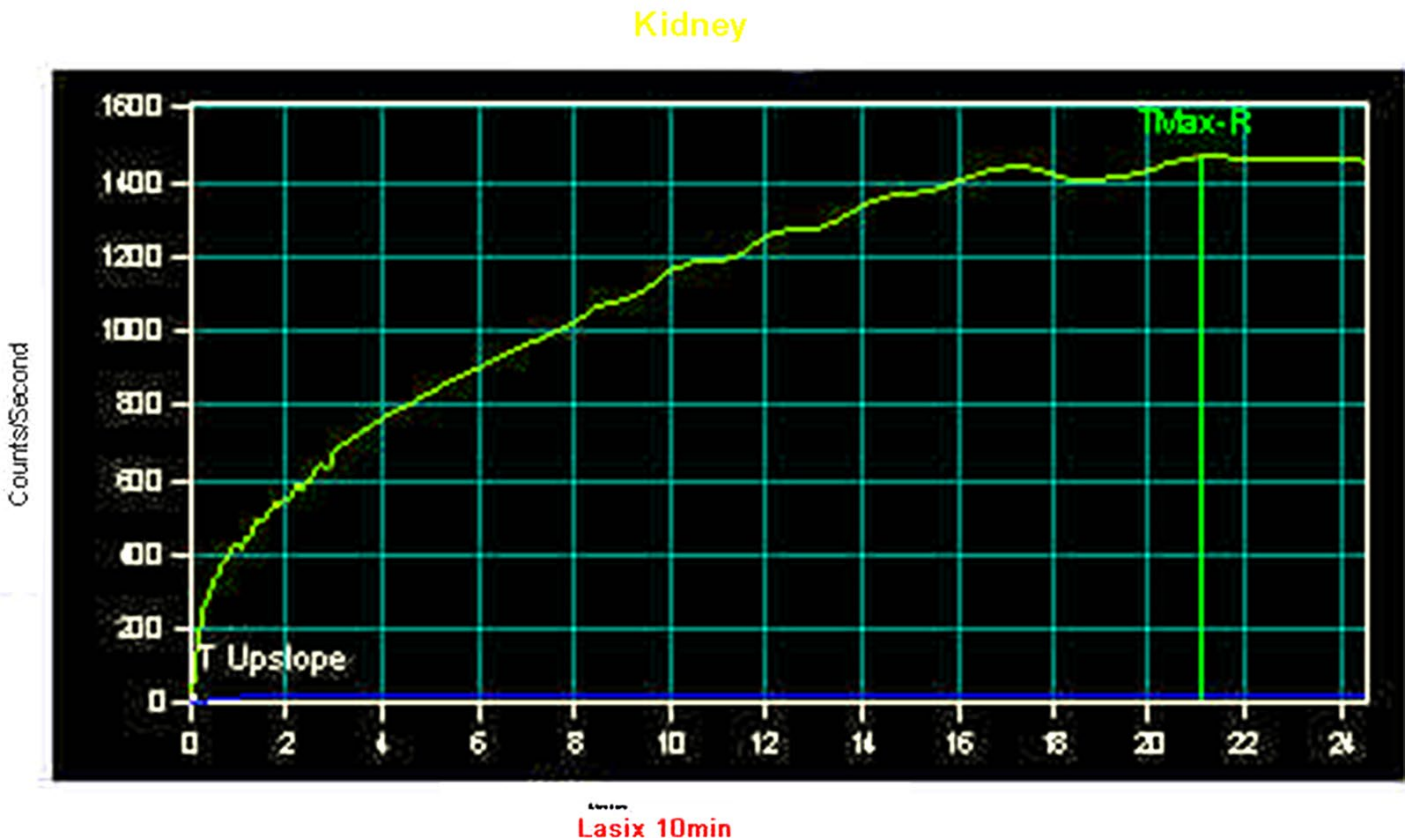
Preoperative renal scintigraphy findings

At six months old, magnetic resonance urography (MRU) and renal scintigraphy revealed HSK with right PUJO (Fig. 1a). The left kidney had a non-functioning pattern, and the right kidney had an obstructive pattern. Renal scintigraphy showed no response to the furosemide loading test (Fig. 2). The patient also showed recurrent urinary tract infection, and his right hydronephrosis had gradually worsened with age (Fig. 1b, c).

**Fig. 3:**
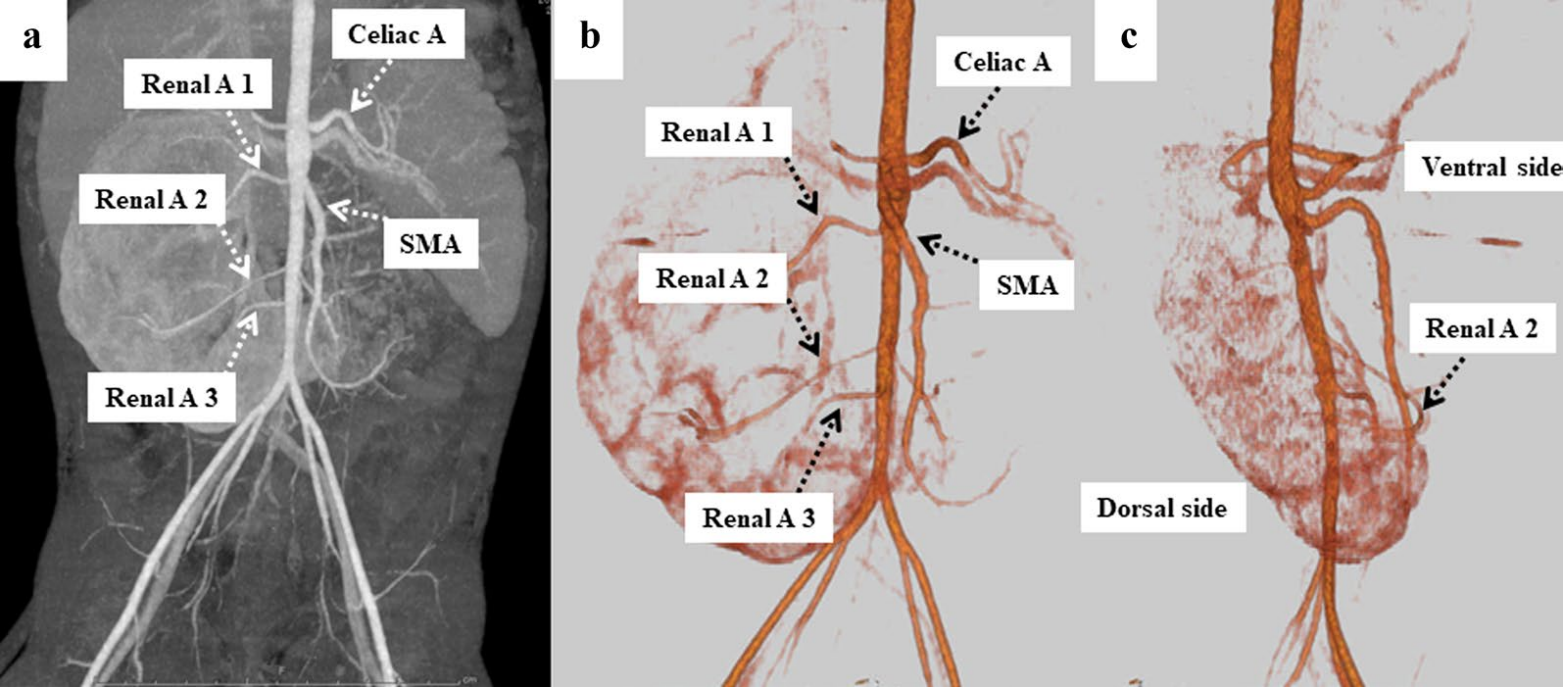
Preoperative enhanced CT findings

Once the patient reached two years old, we decided to perform surgery for the right PUJO to prevent renal dysfunction. Preoperative enhanced computed tomography detected three right renal vessels independently branching from the abdominal aorta (Fig. 3a, b). The middle renal vessels (renal artery 2) were located at the ventral side of the pelvis and were coincident with the site of PUJO (Fig. 3c). We therefore suspected the possibility of extrinsic obstruction due to these CVs in addition to intrinsic stenosis preoperatively.

**Fig. 4:**
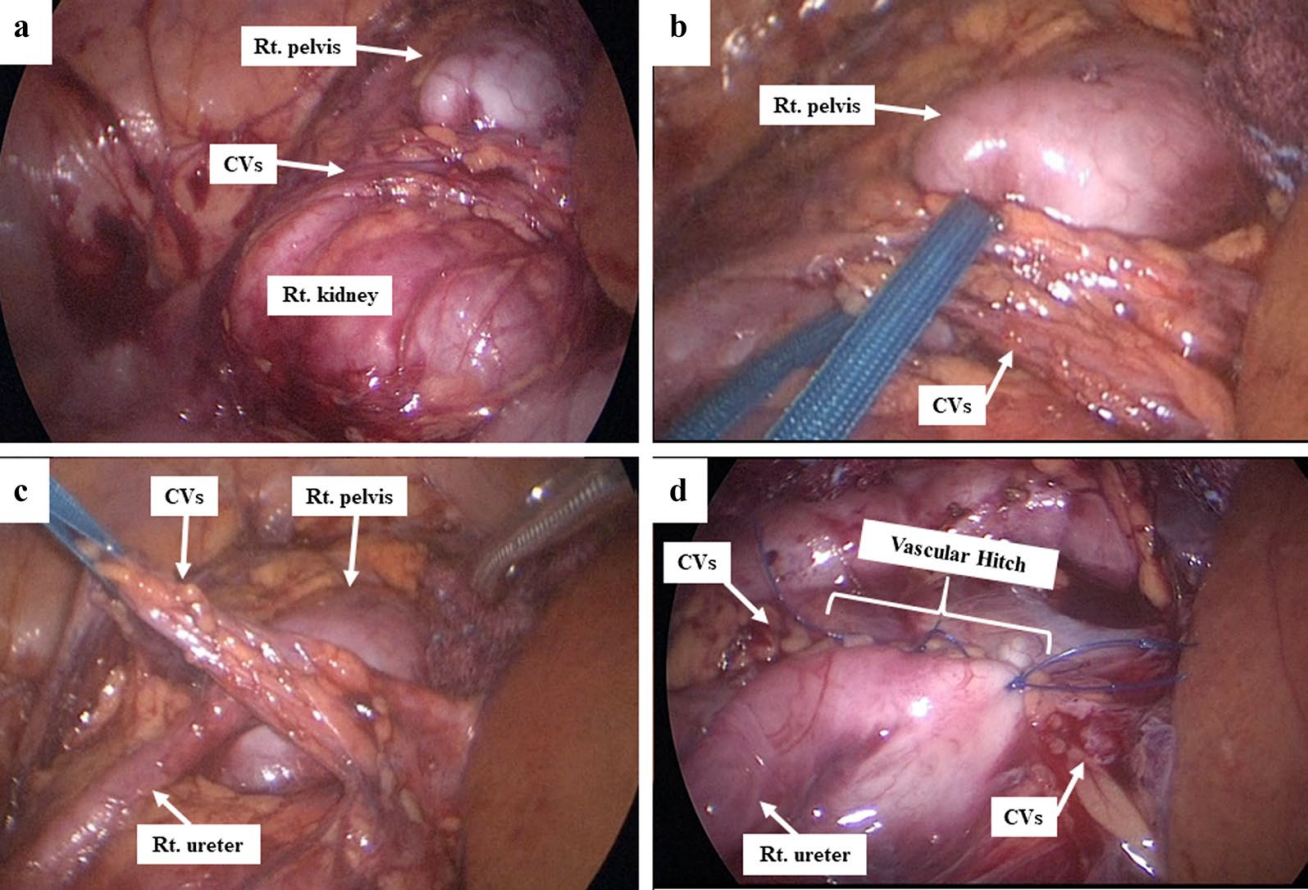
Operative findings and procedures. **a** dilated pelvis and CVs, **b** dissection of CVs, **c** taping of CVs and intraoperative DT with furosemide loading, **d** vascular hitch

The patient underwent laparoscopic surgery electively. Under general anesthesia, the patient was placed in the left semi-lateral position. A 5-mm trocar was inserted at the umbilicus via the open Hasson method for a 5-mm, 30° rigid scope. Pneumoperitoneum was established with 8 mmHg carbon dioxide inflation (4 L/min). Two additional ports were then inserted under laparoscope inspection: a 5-mm port below the xiphoid for the operator’s right hand and a 5-mm port at the middle lower abdomen for the operator’s left hand. The dilated right pelvis and CVs were detected after the ascending colon was dissected and mobilized (Fig. 4a). To confirm the pathogenesis of PUJO, the CVs were dissected and taped with vessel tape (Fig. 4b). After taping the CVs, an intraoperative diuretic test was performed by an anesthesiologist using furosemide loading (Lasix®, 1 mg/kg, Nichi-Iko Pharmaceutical Co., Ltd. Toyama, Japan) (Additional file 1: Video S1). Peristalsis of the right ureter due to urine flowing was recognized after furosemide loading (Fig. 4c). Extrinsic PUJO owing to the CVs was definitively confirmed. We therefore decided to perform transposition for the CVs (VH) instead of AHDP.

The CVs were mobilized in the cranial direction and those were loosely wrapped by dilated pelvic anterior wall using three interrupted sutures of 4–0 non-absorbable monofilament (Fig. 4d). Similar to laparoscopic fundoplication, the wrap construction was made in order to keep the blood perfusion of the CVs. An intra-abdominal drain and stent catheter of the right ureter were not inserted. There were no intraoperative or postoperative complications.

**Fig. 5:**
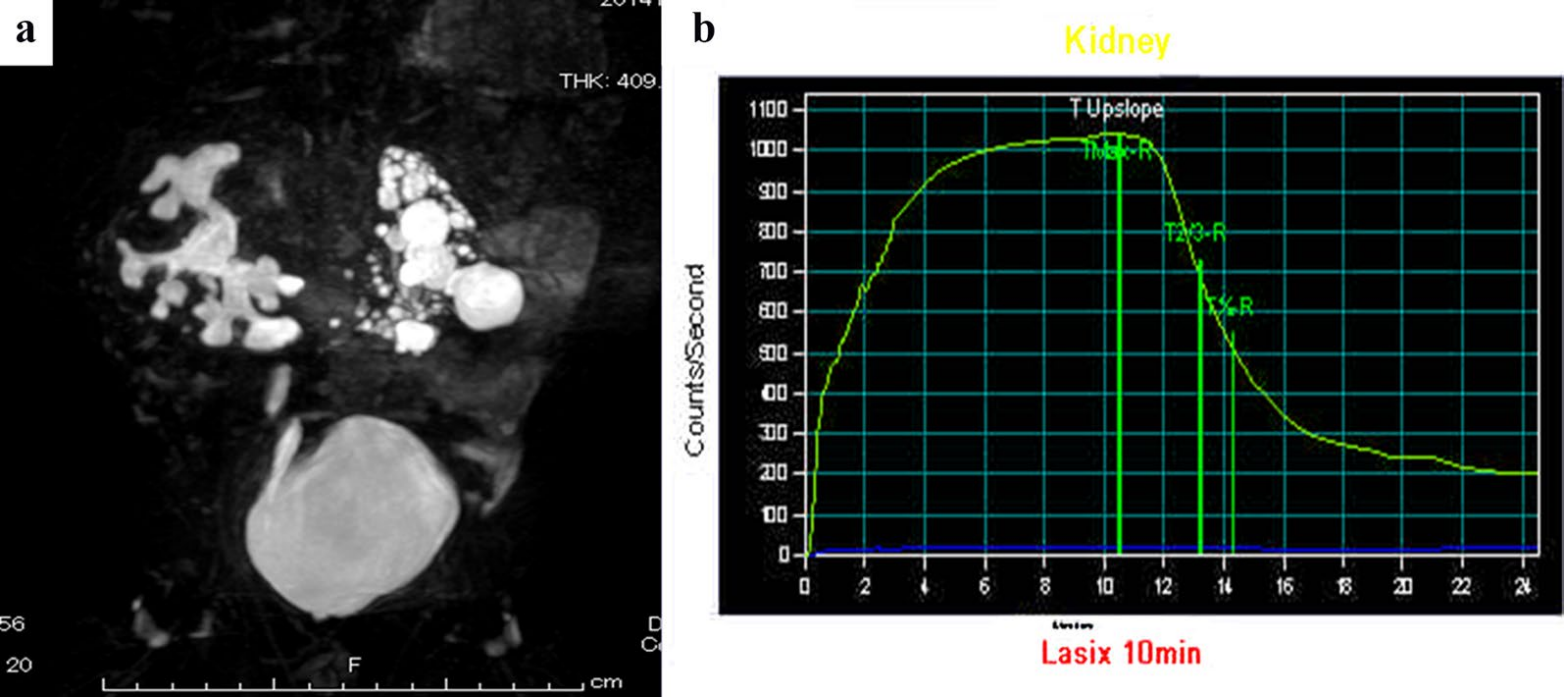
Postoperative MRU and renal scintigraphy findings. **a** MRU, **b** scintigraphy

The post-operative course was uneventful, and MRU showed improvement of the right hydronephrosis (Fig. 5a). In addition, the renal scintigraphy findings improved and showed a favorable response of furosemide loading (Fig. 5b).

## Discussion

PUJO is caused by intrinsic disorganization or by extrinsic compression of CVs. The extrinsic etiology, which induces symptoms in older children and adults, was first reported by Von Rokitansky in 1842 [11]. The CV incidence rate in PUJO has been reported to range from 11 to 15% [12]. However, there is no standard consensus regarding the approach to surgical treatment for this vascular condition.

AHDP for both intrinsic and extrinsic PUJO, developed and reported by Anderson and Hynes, is an established standard procedure [13]. Open AHDP for the treatment of PUJO in children has shown a high success rate and favorable outcomes. Laparoscopic AHDP also reportedly has a high success rate, similar to the open procedure, but resection of the stenotic region and re-anastomosis remain challenging techniques in infants and small children [14]. In addition, uretero-pelvic anastomosis carries a risk of leakage, as well as subsequent urinary peritonitis and postoperative stricture. While anastomosis of AHDP is inevitable for intrinsic PUJO, alternative procedures displacing the CVs cranially and then anchoring them to the anterior wall of dilated pelvis are available for extrinsic PUJO due to the CVs. Chapman modified this procedure by securing more superior side of CVs within a wrap construction [9]. This VH technique was described in a large case series of children in 1999 [10]. The success rate of VH is similar to or higher than that of AHDP with appropriate patient selection, so patient selection is essential for ensuring a successful outcome.

PUJO owing to CVs is not prenatally diagnosed and shows intermittent symptoms. The present case was prenatally diagnosed with right hydronephrosis and left MDCK and carefully followed up. However, the right hydronephrosis gradually worsened, and the patient developed recurrent urinary infection. He ultimately required therapeutic intervention before developing irreversible right renal dysfunction. Based on the preoperative enhanced CT findings, we suspected extrinsic PUJO owing to CVs and performed intraoperative DT. The involvement of intrinsic factors was denied by the confirmation of peristalsis of the right ureter, and laparoscopic VH was successfully performed without PUJ resection or anastomosis. Post-operative complications, such as leakage, urinary peritonitis and postoperative stricture, were avoided.

HSK is a congenital defect of the urinary tract that occurs in 0.25% of the general population and results from abnormal mediastinal fusion [15]. The renal vessels thus develop an abnormal relation to the renal pelvis and ureters. About two-thirds of all HSK cases have an abnormal blood supply to the isthmus. In addition, they have two or three arteries to each kidney arising from the abdominal aorta and one running to the lower pole. HSK possibly result in PUJO of various degree. The ureter usually crosses the anterior surface of the renal isthmus while descending to the bladder. Possible causes of extrinsic PUJO in cases of HSK kidney include a high insertion of the ureter or distortion of the proximal ureter. The proximal ureter loops over the renal isthmus, compression from the aberrant vessels at the hilum. Laparoscopic surgery can provide a clear operative view and clarify the detailed anatomical structure, even in cases of HSK.

## Conclusion

When performing surgery for PUJO, pediatric surgeons must determine the cause of the obstruction and must have technical option of VH with intraoperative DT based on the correct selection of the patients.

## Supplementary Information

**Additional file 1**. Operative procedure of laparoscopic transposition for crossing vessels (vascular hitch).

## Data Availability

The data that support the findings of this study are available from the corresponding author upon reasonable request.

## Abbreviations

PUJO: Pelvic-ureteric junction obstruction
DP: Dismembered pyeloplasty
CVs: Crossing vessels
MDCK: Multicystic dysplastic kidney
HSK: Horseshoe kidney
AHDP: Anderson and Hynes dismembered pyeloplasty
VH: Vascular hitch
MRU: Magnetic resonance urography

## References

[1] Anderson JC, Hynes W (1949). Retrocaval ureter; a case diagnosed pre-operatively and treated successfully by a plastic operation. Br J Urol.

[2] He Y, Song H, Liu P, Sun N, Tian J, Li M (2020). Primary laparoscopic pyeloplasty in children: a single-center experience of 279 patients and analysis of possible factors affecting complications. J Pediatr Urol.

[3] Ramalingam M, Kallappan S, Nachimuthu S (2018). A prospective comparative study of continuous and interrupted suturing in laparoscopic pyeloplasty in 3D Era. J Laparoendosc Adv Surg Tech A.

[4] Ebert KM, Nicassio L, Alpert SA, Ching CB, Dajusta DG, Fuchs ME (2020). Surgical outcomes are equivalent after pure laparoscopic and robotic-assisted pyeloplasty for ureteropelvic junction obstruction. J Pediatr Urol.

[5] Krajewski W, Wojciechowska J, Dembowski J, Zdrojowy R, Szydełko T (2017). Hydronephrosis in the course of ureteropelvic junction obstruction: an underestimated problem? Current opinions on the pathogenesis, diagnosis and treatment. Adv Clin Exp Med.

[6] Polok M, Borselle D, Toczewski K, Apoznański W, Patkowski D (2019). Detection rate of crossing vessels in pediatric hydronephrosis: Transperitoneal laparoscopy versus open lumbotomy. Adv Clin Exp Med.

[7] Wong MCY, Piaggio G, Damasio MB, Molinelli C, Ferretti SM, Pistorio A (2018). Hydronephrosis and crossing vessels in children: Optimization of diagnostic-therapeutic pathway and analysis of color Doppler ultrasound and magnetic resonance urography diagnostic accuracy. J Pediatr Urol.

[8] Hellstrom J, Giertz G, Lindblom K (1951). Pathogenesis and treatment of hydronephrosis. J Belge Urol.

[9] Chapman TL (1959). Urology in outline.

[10] Presce D, Campobasso P, Costa L, Battaglino F, Musi L (1999). Ureterovascular hydronephrosis in children: is pyeloplasty always necessary?. Eur Urol.

[11] Von Rokitansky CF (1842). Handbuch der Pathologischen Anatomie.

[12] Singh RR, Govindarajan KK, Chandran H (2010). Laparoscopic vascular relocation: alternative treatment for renovascular hydronephrosis in children. Pediatr Surg Int.

[13] Cao H, Zhou H, Liu K, Ma L, Liu D, Tao T (2016). A modified technique of paraumbilical three-port laparoscopic dismembered pyeloplasty for infants and children. Pediatr Surg Int.

[14] Leung L, Chan IH, Chung PH, Lan LC, Wong KK, Tam PK (2016). Outcomes of Re-Intervention for Laparoscopic Transperitoneal Pyeloplasty in Children. J Laparoendosc Adv Surg Tech A.

[15] Bleve C, Bucci V, Conighi ML, Battaglino F, Costa L, Fasoli L, et al. Horseshoe kidney and uretero-pelvic-junction obstruction in a pediatric patient. Laparoscopic vascular hitch: a valid alternative to dismembered pyeloplasty. Pediatr Med Chir. 2017; 13;39(4): 178. 10.4081/pmc.2017.178 29502388

